# Mortality risk prediction in high-risk patients undergoing coronary artery bypass grafting: Are traditional risk scores accurate?

**DOI:** 10.1371/journal.pone.0255662

**Published:** 2021-08-03

**Authors:** Maxim Goncharov, Omar Asdrúbal Vilca Mejia, Camila Perez de Souza Arthur, Bianca Maria Maglia Orlandi, Alexandre Sousa, Marco Antônio Praça Oliveira, Fernando Antibas Atik, Rodrigo Coelho Segalote, Marcos Gradim Tiveron, Pedro Gabriel Melo de Barros e Silva, Marcelo Arruda Nakazone, Luiz Augusto Ferreira Lisboa, Luís Alberto Oliveira Dallan, Zhe Zheng, Shengshou Hu, Fabio Biscegli Jatene

**Affiliations:** 1 Department of Cardiovascular Surgery, Instituto do Coração, University of São Paulo, São Paulo, Brazil; 2 Department of Cardiovascular Surgery, Hospital Samaritano Paulista, São Paulo, Brazil; 3 Department of Cardiovascular Surgery, Beneficência Portuguesa de São Paulo, São Paulo, Brazil; 4 Department of Cardiovascular Surgery, Instituto de Cardiologia do Distrito Federal, Brasília, Brazil; 5 Department of Cardiovascular Surgery, Instituto Nacional de Cardiologia do Rio de Janeiro, Rio de Janeiro, Brazil; 6 Department of Cardiovascular Surgery, Hospital Santa Casa de Misericórdia de Marília, Marília, Brazil; 7 Department of Cardiovascular Surgery, Hospital de Base de São José do Rio Preto, São José do Rio Preto, Brazil; 8 Department of Cardiovascular Surgery, Fuwai Hospital, Beijing, China; Case Western Reserve University School of Medicine, UNITED STATES

## Abstract

**Background:**

The performance of traditional scores is significantly limited to predict mortality in high-risk cardiac surgery. The aim of this study was to compare the performance of STS, ESII and HiriSCORE models in predicting mortality in high-risk patients undergoing CABG.

**Methods:**

Cross-sectional analysis in the international prospective database of high-risk patients: HiriSCORE project. We evaluated 248 patients with STS or ESII (5–10%) undergoing CABG in 8 hospitals in Brazil and China. The main outcome was mortality, defined as all deaths occurred during the hospitalization in which the operation was performed, even after 30 days. Five variables were selected as predictors of mortality in this cohort of patients. The model’s performance was evaluated through the calibration-in-the-large and the receiver operating curve (ROC) tests.

**Results:**

The mean age was 69.90±9.45, with 52.02% being female, 25% of the patients were on New York Heart Association (NYHA) class IV and 49.6% had Canadian Cardiovascular Society (CCS) class 4 angina, and 85.5% had urgency or emergency status. The mortality observed in the sample was 13.31%. The HiriSCORE model showed better calibration (15.0%) compared to ESII (6.6%) and the STS model (2.0%). In the ROC curve, the HiriSCORE model showed better accuracy (ROC = 0.74) than the traditional models STS (ROC = 0.67) and ESII (ROC = 0.50).

**Conclusion:**

Traditional models were inadequate to predict mortality of high-risk patients undergoing CABG. However, the HiriSCORE model was simple and accurate to predict mortality in high-risk patients.

## Introduction

Over time, cardiovascular surgery results have progressively improved. One of the reasons for the improvement is associated with the risk stratification brought by risk scores and perioperative optimization [1]. In a continuous search for excellence, the application of ever more accurate score models is essential, especially in the evaluation of indications for new cost-effective procedures [2]. In addition, it is necessary to fully inform each patient about the risks associated with this procedure, which should be adjusted to the results of the hospital [3].

In this scenario, several models have been built and validated, aiming to reach more accurate predictions for specific populations. Among these models, STS [4] and ESII [5] reverberate the most and, at the same time, are supported by international guidelines. Both are recommended for patients undergoing most cardiovascular procedures. STS’ greatest advantage over ESII is probably the sample size, which is updated periodically. At the same time, one of the biggest criticisms of this voluntary registry may be related to estimated values unreachable for other populations [6]. It is known that the results of mandatory registries, which include all operated patients, can have high deviation [7]. In underdeveloped or developing countries, the evaluation provided by these tools can be impaired, due to the measurement of only part of the treatment, not the health system [8].

Even with the improvement of registry systems and refinement in formulation techniques of those tools, predictions are still impaired, especially in the high-risk subgroup [9, 10]. This may be related to the small number of high-risk patients included in the registries that originated the traditional models. In this aspect, traditional models would be important for a first categorization (approximation), but not for defining exactly what happens to patients at higher risk of death after cardiac surgery, as supported by the evidence. Therefore, this new model would be a second step and would come to a more accurate decision-making, through the recalibration and remodeling of variables for the high-risk population.

In this scenario, we evaluated the performance of STS, ESII and the HiriSCORE model derived from high-risk patients undergoing CABG (https://clinicaltrials.gov/ct2/show/NCT02560285).

## Methods

### Ethics and consent form

This study is a sub-analysis belonging to the project entitled "High-risk Patients in Cardiac Surgery Procedures: HiriSCORE”, registered online under number 13795, submitted and approved by the HCFMUSP Ethics Commission for Analysis of Research Projects (CAPPesq) as SDC: 4256/15/083.

### Sample

The cross-sectional analysis is part of the HiriSCORE Project and coordinated by the Cardiac Surgery department of "Instituto do Coração do Hospital das Clínicas da Faculdade de Medicina da Universidade de São Paulo" (InCor-HCFMUSP).

All cases were consecutively operated from April 2016 to August 2019. Data came from 8 hospitals in Brazil (7) and China (1):

1. Instituto do Coração, HCFMUSP, São Paulo, SP, Brazil.
2. Hospital de Base de São José do Rio Preto, SP, Brazil.
3. Instituto Nacional de Cardiologia do Rio de Janeiro, RJ, Brazil.
4. Fuwai Hospital, Beijing, China.
5. Hospital Beneficência Portuguesa, SP, Brazil.
6. Hospital Santa Casa de Marilia, SP, Brazil.
7. Hospital Samaritano Paulista, SP, Brazil.
8. Instituto de Cardiologia do Distrito Federal, DF, Brazil.

**Fig 1:**
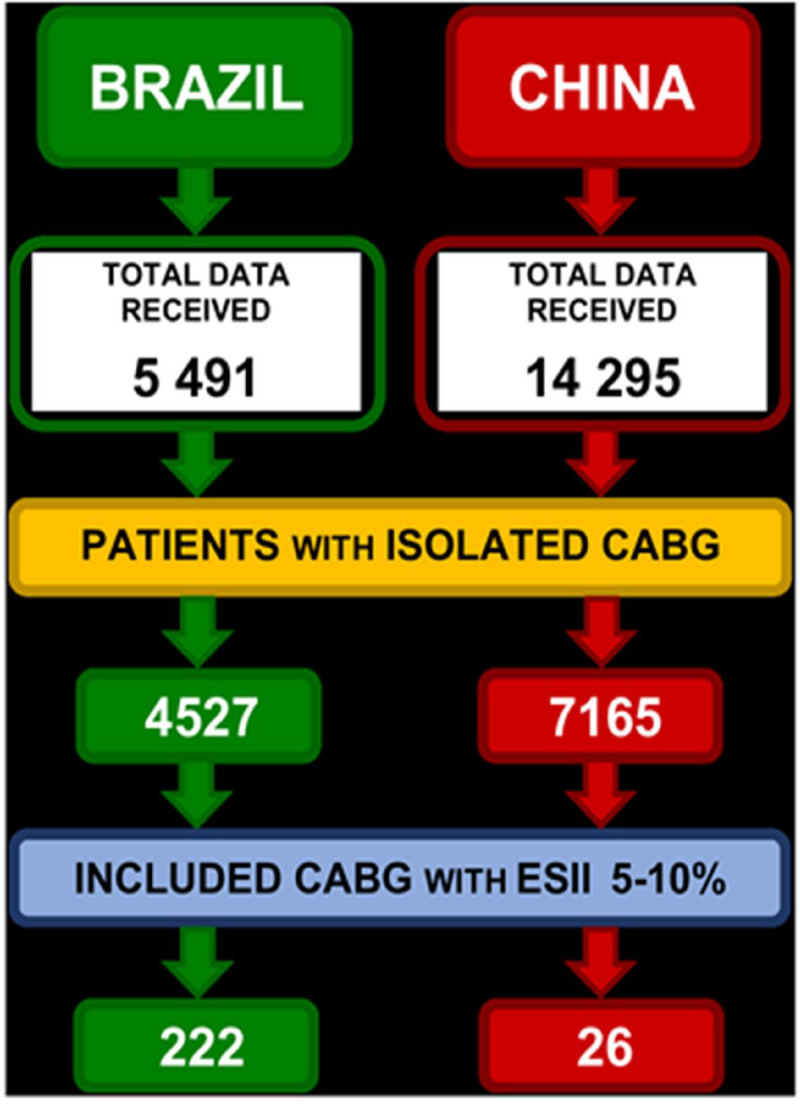
Flowchart of selection and recruitment of high-risk patients undergoing CABG—HiriSCORE database, 2019.

The total sample consisted of 19,786 patients who underwent CABG, 11,692 of whom underwent isolated CABG. For this analysis, we have selected 248 patients considered at high risk (Fig 1). The final cohort included 248 patients (222 patients from Brazil and 26 patients from Fuwai Hospital in China) who underwent CABG with a mortality risk of 5 to 10% predicted by ESII (S1 Table).

### Inclusion and exclusion criteria

#### Inclusion criteria

Patients aged 18 years or older who underwent isolated CABG and with mortality expected by EuroSCORE II between 5 and 10%

#### Exclusion criteria

Patients undergoing any other cardiac procedures than CABG. To have a more homogeneous sample for the construction of the HiriSCORE model, 4 patients with EuroSCORE II > 10% were excluded.

There were no cases of MIDCAB or OPCAB in the studied sample.

### Collecting, defining and organizing data

This analysis was made in the HiriSCORE Project database (https://clinicaltrials.gov/ct2/show/NCT02560285). It is a prospective multicenter and international registry. Data were collected by people trained for this purpose in each participating center and incorporated online on the REDCap platform (https://redcap.hc.fm.usp.br) through three interfaces available online: preoperative, intraoperative, and post-operative. Truthfulness and completeness of data were supervised by the registry’s executive committee. Variable definitions were adopted to respond to ESII (http://euroscore.org/calc.html) and STS (http://riskcalc.sts.org/stswebriskcalc/#/calculate) calculations. The outcome analyzed was mortality, defined as all deaths that occurred during the hospitalization in which the operation was performed, even after 30 days.

### Statistical analysis

The analysis was performed using the statistical software STATA version 13.1 (StataCorp, Texas, USA) for MacOS. To assess the distribution of the data, the Shapiro-Wilk test and homogeneity between groups were performed. Continuous variables were expressed as mean ± standard deviation and categorical variables as absolute and relative frequencies. The association between mortality and the predictors was verified by the Chi-square and Fisher’s exact tests for the contingency tables. For the prediction of in-hospital mortality, stepwise multivariate logistic regression analysis was verified. The elaboration of the HiriSCORE was performed using stepwise multivariate logistic regression, with the pre- and intra-operative predictors, in which the risk value (OR) may vary according to the sum of variables that represent risk. All variables with inclusion criteria in the score were described in the results and expressed in a table with a coefficient value, 95% CI and p-value. The performance of the ESII and STS models was measured by comparing the mortality observed in the current study with the mortality predicted by the models in the established risk groups. Therefore, to assess the ability of ESII and STS to identify the risk of individuals included in the current study, the calibration-in-the-large curve and the discrimination by area under the ROC curve were performed. P-values of <0.05 were considered significant and plausible variables were identified as predictors of mortality after cardiac procedures. For internal validation of the HiriSCORE model, the method of discrimination by area under the ROC curve and calibration-in-the-large was selected.

## Results

Overall, Table 1 includes data of 248 patients who underwent CABG surgery.

**Table 1:** Description of variables used in high-risk CABG database.

Variables	Total (n = 248)		95% CI
	n	%	
**Age (years)**	69.90 ± 9.45		68.55–70.90
**Body mass index (kg/m**^**2**^**)**	26.11 ± 4.55		25.50–26.60
**Systolic pulmonary artery pressure (mmHg)**	27.30 ± 13.30		25.70–29.00
**Left ventricle ejection fraction (%)**	46.00 ± 14.80		43.90–47.60
**Glucose (mg/dL)**	152.00 ± 78.00		140.7–161.16
**Creatinine (mg/dL)**	1.52 ± 1.10		1.37–1.64
**Gender (male)**	119	47.98	0.42–0.54
**Hypertension**	212	85.48	0.80–0.90
**Peripheral arterial disease**	49	19.76	0.15–0.25
**Dialysis**	4	1.61	0.004–0.4
**Cerebrovascular disease**	73	29.44	0.24–0.36
**Pre-operative intra-aortic balloon pump**	50	20.16	0.15–0.26
**Pulmonary hypertension**	144	58.06	0.51–0.64
**Diabetes mellitus**	143	57.66	0.51–0.63
**Insulin control**	80	32.26	0.26–0.38
**COPD**	59	23.79	0.19–0.29
**Atrial fibrillation:**			
**Paroxysmal/persistent**	16	6.45	0.05–0.13
**Continued/permanent**	5	2.02	
**Previous cardiac intervention:**			
**Previous PCI**	43	17.34	0.13–0.23
**Previous CABG**	9	3.63	0.02–0.07
**Previous valve surgery**	4	1.61	0.004–0.4
**Previous MI**	184	74.19	0.69–0.79
**Three-vessel coronary artery disease**	201	81.05	0.76–0.86
**Left main stenosis > 50%**	75	30.24	0.25–0.36
**NYHA IV**	63	25.40	0.49–0.62
**CCS 4**	123	49.60	0.43–0.56
**Moderate heart valve disease**	90	36.29	0.30–0.42
**Urgency or emergency status**	212	85.48	0.80–0.89
**Death**	33	13.31	0.09–0.18

CABG: coronary artery bypass grafting; CCS: Canadian Cardiovascular Society; COPD: chronic obstructive pulmonary disease; MI: myocardial infarction; NYHA: New York Heart Association; PCI: percutaneous coronary intervention.

The average age was 69.9±9.45y (95% CI 68–70). Most patients were female (52%). There was a high prevalence of prior myocardial infarction (74%), hypertension (85%) and urgent or emergency surgery (85%). Eighty-one percent of patients had three-vessel coronary artery disease. Almost half (49%) of the patients had unstable angina (CCS 4). The observed mortality reached 13%.

In the association analysis, all variables with a focus on preoperative variables were studied. The main variables of interest are shown in Table 2.

**Table 2:** Association analysis in the high-risk CABG database.

Association	Deaths (n = 33)		Alive patients (n = 215)		p
	n	%	n	%	
**Male gender**	16	13.4	103	86.6	0.951
**Female gender**	17	13.2	112	86.8	
**Hypertension**	25	11.8	187	88.2	0.88
**Peripheral artery disease**	5	10.2	44	89.8	0.475
**Dialysis**	0	0	4	100	0.563
**Cerebrovascular disease**	5	6.8	68	93.2	0.053
**Pulmonary hypertension**	17	11.8	127	88.2	0.413
**Diabetes mellitus**	13	9.1	130	90.9	0.023
**Insulin-dependent**	5	6.3	75	93.8	0.024
**Without atrial fibrillation**	28	12.3	199	87.7	0.128
**Persistent/paroxysmal atrial fibrillation**	4	25	12	75	
**Continued/permanent atrial fibrillation**	1	20	4	80	
**COPD**	9	15.3	50	84.7	0.614
**Previous PCI**	2	4.7	41	95.3	0.047
**Previous CABG**	3	33.3	6	66.7	0.103
**Previous valve procedure**	0	0	4	100	0.563
**Previous MI**	22	12	162	88	0.289
**Coronary disease**	32	13.2	211	86.8	0.656
**Three-vessel coronary artery disease**	30	14.9	171	85.1	0.121
**Left main disease > 50%**	16	21.3	59	78.7	0.014
**NYHA I**	11	20	44	80	0.323
**NYHA II**	4	10.5	34	89.5	
**NYHA III**	7	9.3	68	90.7	
**NYHA IV**	10	15.9	53	84.1	
**CCS 4**	14	11.4	109	88.6	0.376
**Moderate heart valve disease**	18	20	72	80	0.019
**Urgency/emergency status**	31	14.6	181	85.4	0.139
**Emergency**	2	40	3	60	0.188
**Pre-operatory IABP**	10	20	40	80	0.119
Body mass index > 30 kg/m^2^	9	25.7	26	74.3	0.02
Creatinine clearance < 30 mL/min/m^2^	10	30.3	23	69.7	0.002

CABG: coronary artery bypass grafting; CCS: Canadian Cardiovascular Society; COPD: chronic obstructive pulmonary disease; IABP: intra-aortic balloon pump; MI: myocardial infarction; NYHA: New York Heart Association; PCI: percutaneous coronary intervention.

In the association analysis in the cohort selected to prepare the HiriSCORE model, the following variables were related to deaths: diabetes (p = 0.023); insulin-dependent diabetes (p = 0.024); lesion of the left main coronary artery > 50% (p = 0.014); moderate heart valve disease (p = 0.019); body mass index > 30 kg/m^2^ (p = 0.02) and creatinine clearance <30 mL/min (p = 0.002) (Table 2). These variables were selected for stepwise multivariate regression analysis to create the HiriSCORE model.

### Elaboration of HiriSCORE model

After association analysis with subsequent multivariate logistic regression using data of 248 patients with predicted risk by ESII 5–10% undergoing CABG, 5 significant variables were determined: body mass index, creatinine clearance, left main coronary artery stenosis, moderate heart valve disease and glucose (Table 3).

**Table 3:** Elaboration of HiriSCORE model.

Variables	Coef.	95% CI	p
**Body mass index > 30 kg/m**^**2**^	1.223577	0.26–2.18	0.013
**Creatinine clearance < 30 mL/min/m**^**2**^	1.014661	0.08–1.94	0.033
**Left main coronary artery stenosis > 50%**	0.9135525	0.1–1.7	0.026
**Moderate heart valve disease**	1.098947	0.27–1.92	0.009
**Glucose > 150 mg/dL**	0.8668853	0.02–1.7	0.044
**Constant**	−3.623276	−4.6–−2.6	0.000

The HiriSCORE risk calculator was developed based on the variables described above. The logistical formula for calculating the mortality risk is as follows:

$$RDM=Exp\;(\beta_{0}+\Sigma \;\beta_{i}x_{i})/\left[1+Exp\;\left(\beta_{0}+\Sigma \;\beta_{i}x_{i}\right)\right]$$

where RDM is the mortality risk, Exp is the exponential function, β_0_ is the constant with value -3.623276, β_i_ is the tabulated coefficient of the independent variable x_i_.

### Performance validation of ESII, STS and HiriSCORE models

#### Calibration of ESII, STS and HiriSCORE models

Table 4 shows that both traditional risk models underestimate mortality in high-risk patients, when the HiriSCORE model showed good performance in all 5 subgroups.

**Table 4:** Comparative evaluation of the calibration-in-the-large of ESII, STS and HiriSCORE models.

Quintiles	ESII		STS		HiriSCORE	
	Observed	Predicted	Observed	Predicted	Observed	Predicted
1	14	5.2	12	0.8	3.7	4.18
2	14	5.61	2	1.3	8.51	7.07
3	12.24	6.4	4.08	1.6	10	12.6
4	12	7.2	21.57	2.2	20.41	16.5
5	14.29	8.81	27.08	3.9	31.71	35.19
Mean	13.306	6.644	13.346	1.96	14.866	15.108
O−P	6.662		11.386		−0.242	

O−P: observed minus predicted.

**Fig 2:**
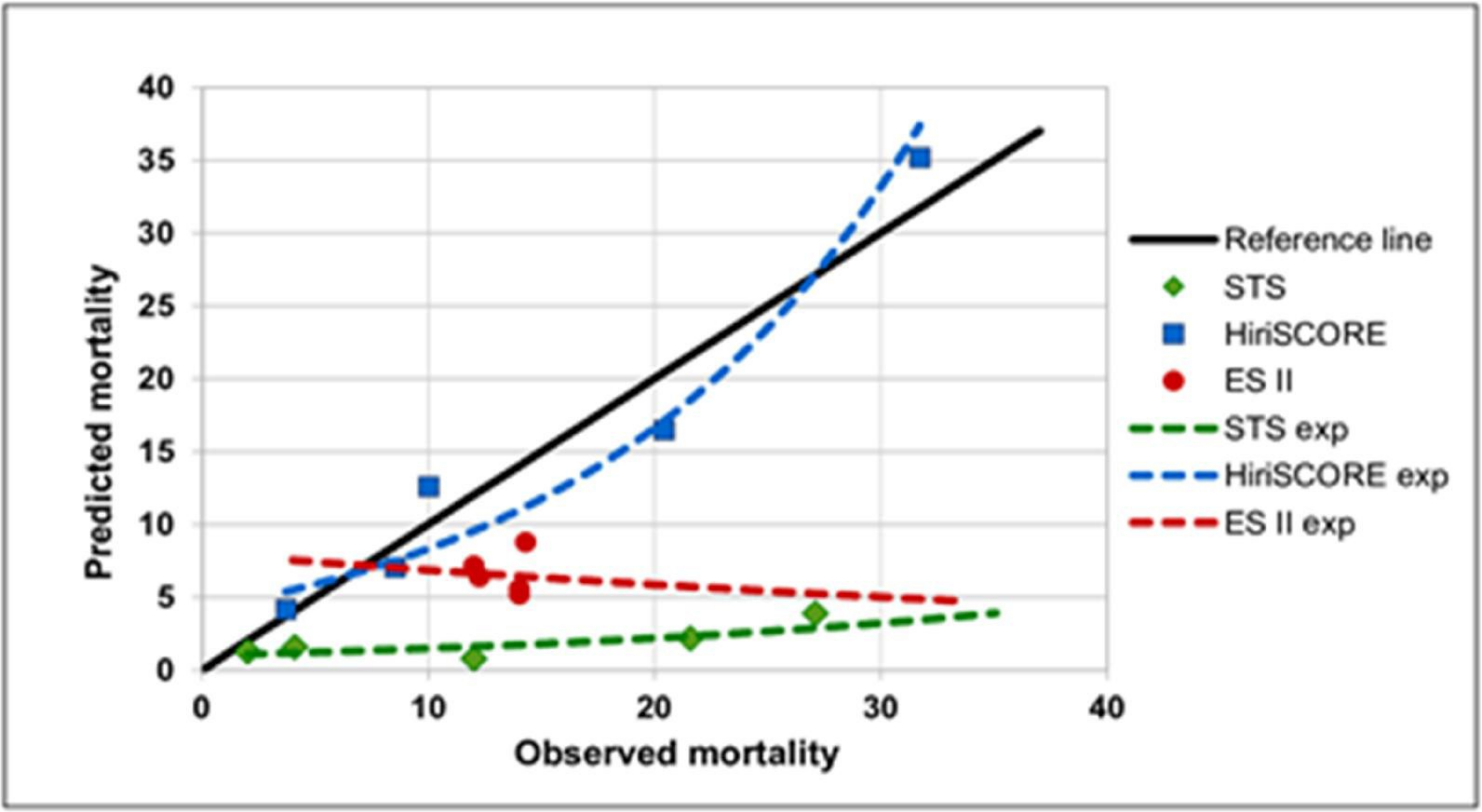
Calibration-in-the-large for ESII, STS and HiriSCORE for high-risk CABG.

The expected mean mortality in the established groups (quintiles) by the HiriSCORE model was 15.0%, by the STS was 2.0% and by the ESII was 6.6% (p <0.05). The expected mortality by ESII was > 3 times that of STS and < 2 times that of HiriSCORE (Fig 2).

The calibration-in-the-large showed that the expected mortality by STS (observed minus predicted [O-P] = 11.4) and ESII (O−P = 6.6) underestimated the mortality observed in high-risk patients undergoing CABG. However, the HiriSCORE model showed good calibration (O−P = −0.2).

### Discrimination for ESII, STS and HiriSCORE models

**Fig 3:**
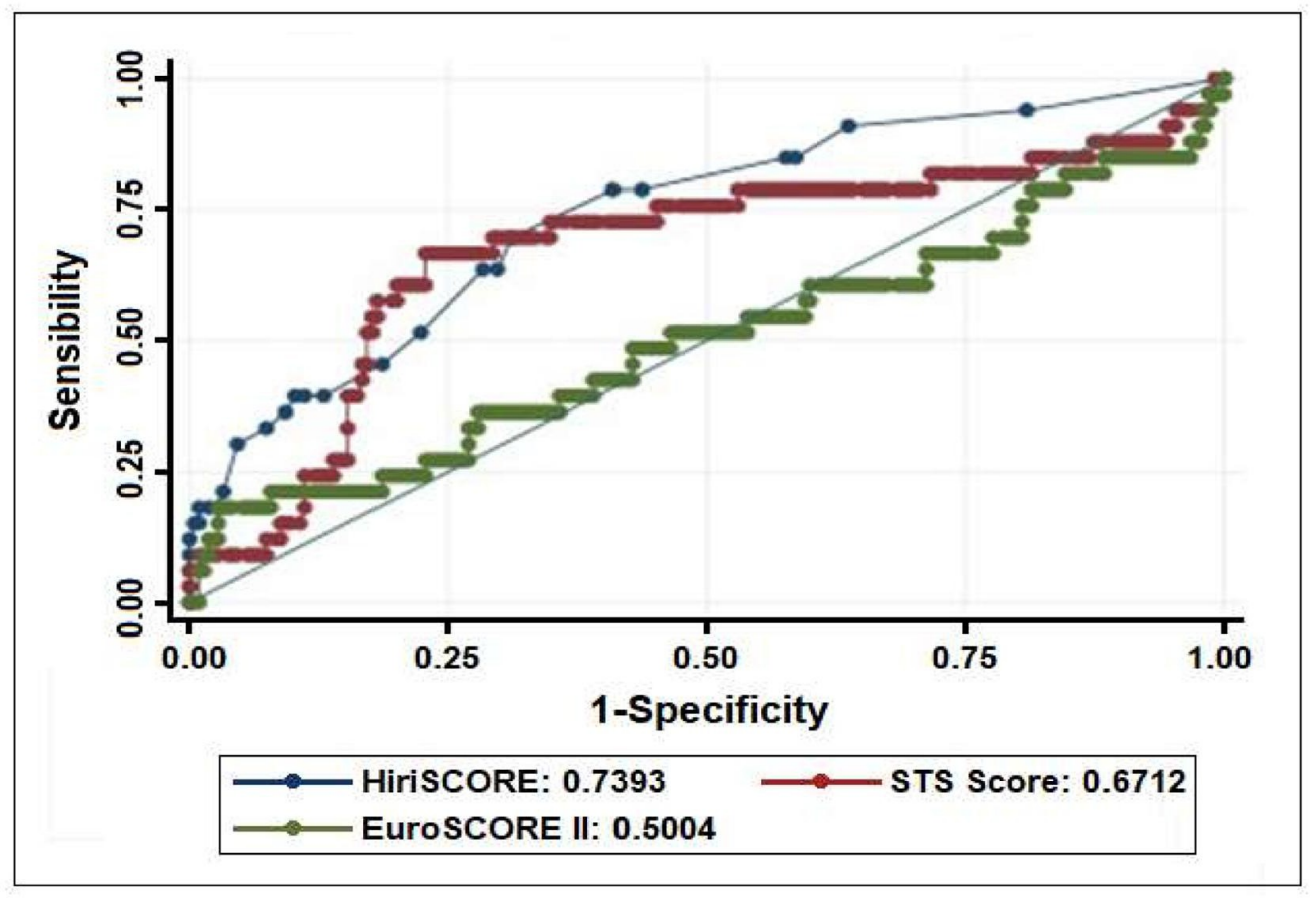
ROC curve for ESII, STS and HiriSCORE models for high-risk CABG.

As for discrimination, HiriSCORE model showed a satisfactory result of an area under the ROC curve (AUC) of 0.74 (95% CI 0.64–0.82). Analyzing traditional risk scores, we found that STS was better than ESII, obtaining a limit value of 0.67 (95% CI 0.62–0.72) against 0.50 (95% CI 0.58–0.69) of the AUC. It is important to emphasize that the literature considers good clinical models when the AUC is > 0.7 (Fig 3).

The HiriSCORE model showed a good AUC (0.74) while the STS and EuroSCORE II were poor in predicting mortality in high-risk patients undergoing CABG. Therefore, traditional scores were ineffective in discriminating mortality in high-risk patients.

## Discussion

To date, there are no studies that assess the prediction of mortality risk in specific high-risk patients undergoing CABG. To estimate risk is to quantify complications that may occur after surgical procedures, allowing a better team planning, as well as decision-making and behavior regarding the procedures.

The two models with the most widespread use for cardiovascular surgery are STS and ESII. In addition, dissonant results appear throughout literature when applied in different scenarios [11–13]. However, evidence shows that, in patients considered to be at high risk for cardiac surgery, traditional scores lose accuracy in predicting mortality [14, 15].

This is probably because risk models originate from general populations of cardiac surgery patients, where most of them present low and medium risk and few are determined to be at high risk, especially in CABG surgeries [16]. For this scenario, procedure-specific models can be a solution for more accurate risk estimation for high-risk groups [17, 18]. Although the predictive variables for mortality after cardiac surgery are always the same, the most important is the weight of the coefficient given to each variable in relation to the specific outcome and group of patients. This is related to the degree of calibration of a model during the validation test. Therefore, calibration becomes the central phase for the validation of a risk prediction model, as it assesses how close the variable is to the outcome for such a scenario [18]. Thus, even with only a few variables, an instrument can be potential to predict mortality [19] and improve planning.

Perhaps the best idea for this scenario was launched by Ranucci in 2016 [20], adopting a two-stage approach for high-risk patients. Here, the high-risk population was underestimated by EuroSCORE II, therefore, patients with mortality risk above 25% were re-evaluated with more than 4 variables.

In this study, 5 predictors were defined. These predictors are partially involved in both, ESII and STS models, but with different coefficients. It shows the importance of reviewing the indication and re-stratifying patients with diabetes, overweight, renal dysfunction, significant left main coronary artery stenosis and moderate heart valve disease in patients referred for CABG.

Regarding the existing evidence in traditional models, Nashef et al [21] conducted a multicentric study that aimed to validate ESII using the STS database, and found similar observed and expected mortality, along with 0.77 in the model’s discriminative capability, reaching what the authors concluded to be an accurate result. Sergeant P. et al. [22] analyzed CABG patients using ESII, and the model reached a discriminative performance of 0.83, offering a good performance, but overestimating the risk in low-risk patients and underestimating it in high-risk patients. Yamaoka et al. [23] compared ESII to STS in the same sample and concluded that, regarding risk calibration, ESII was better, while STS overestimated risks. In this study, in the highest risk quartile, ESII showed excellent calibration and discrimination performance. In our study, calibration of the ESII was better than the STS, but discrimination was worse. One explanation can be the specific type of procedure evaluated.

Schrutka et al. reported the discriminatory power of scoring systems in patients treated with extracorporeal membrane oxygenation (ECMO) following cardiac surgery: both STS and ESII models showed low discriminatory accuracy in high-risk cohort [24]. The first Brazilian validation of ESII is one study that ratifies our findings [25], where ESII was not well calibrated and underestimated the risk in high- and low-risk patients.

In 2020, Hu et al. performed a risk model for CABG patients based on the Chinese Cardiac Surgery Registry (CCSR) [26]. Compared with ESII and SinoSCORE, the CCSR model had better discrimination and calibration. As we can see, it includes 80% of the HiriSCORE variables.

Following this trend, we have compared the performance of HiriSCORE model to predict mortality in high-risk patients undergoing CABG. In this paper, we evaluated the performance of ESII and STS and compared them with the HiriSCORE model. Here, the predicted mortality by the STS was 2% and by the ESII was 6.6%, for the overall 13.3% observed mortality. The reclassification of patients leads to a change in medical concept about the best treatment strategy for the patient, considering alternatives such as percutaneous intervention or medical treatment. The better stratified patients, the greater the impact on medical practice.

Although for CABG the use of STS is still more recommended than ESII [27], our results disagree with its use in high-risk patients. Over time, the severity of patients referred for CABG increased [27], so it is essential to build and validate more accurate prediction models for planning, in addition to an open and transparent discussion with patients and family members.

In addition, Shahian et al. in 2018 reported that a limitation of the score is that STS database does not have data on high-risk patients [28]. On the other hand, the ESII database presented problems because up to 43% of patients presented biased information related to mortality [29].

Therefore, the presence of body mass index > 30 kg/m^2^, glucose > 150 mg/dL, creatinine clearance <30 mL/min/m, left main coronary artery stenosis and moderate heart valve disease become the simplest and best way to predict mortality in high-risk patients undergoing CABG.

### Study limitations

First, as an international multicenter observational record, HiriSCORE project database had to be organized to avoid selection or definition bias. Therefore, the consolidated data had to be evaluated in relation to the consistency, accuracy, and completeness of the information, as well as monitoring the inclusion of patients and following up on the results. Second, ESII was used as an inclusion criterion for high-risk patients in the study. This may have influenced the results. However, the influence would be more in favor than against this model, and that is not what happened, which can further reinforce the evidence found. Third, it would be the 30-day mortality data of the discharged patients, since although the list was delivered by the participating hospitals, we were unable to check with the death verification system at the reference sites. Even understanding that complications in high-risk patients usually happen immediately after surgery.

## Conclusion

The HiriSCORE model for high-risk patients undergoing CABG was better than STS and ESII. We encourage external validation of this model to be used by heart teams as an aid in making better strategy decisions in patients considered to be at high risk for CABG.

## Supporting information

S1 Table Characteristics of the final sample of patients from Brazil and China submitted to isolated CABG with ESII between 5–10%. (DOCX)
