# Correction: Mortality risk prediction in high-risk patients undergoing coronary artery bypass grafting: Are traditional risk scores accurate?

**DOI:** 10.1371/journal.pone.0258706

**Published:** 2021-10-12

**Authors:** Maxim Goncharov, Omar Asdrúbal Vilca Mejia, Camila Perez de Souza Arthur, Bianca Maria Maglia Orlandi, Alexandre Sousa, Marco Antônio Praça Oliveira, Fernando Antibas Atik, Rodrigo Coelho Segalote, Marcos Gradim Tiveron, Pedro Gabriel Melo de Barros e Silva, Marcelo Arruda Nakazone, Luiz Augusto Ferreira Lisboa, Luís Alberto Oliveira Dallan, Zhe Zheng, Shengshou Hu, Fabio Biscegli Jatene

The initials of the third, sixth, eighth, ninth, tenth, eleventh, twelfth, thirteenth, and sixteenth authors are indexed incorrectly in PubMed. The correct initials are, respectively: Arthur CPS; Oliveira MAP; Segalote RC; Tiveron MG; de Barros e Silva PGM; Nakazone MA; Lisboa LAF; Dallan LAO; and Jatene FB.

The correct citation is: Goncharov M, Mejia OAV, Arthur CPS, Orlandi BMM, Sousa A, Oliveira MAP, et al. (2021) Mortality risk prediction in high-risk patients undergoing coronary artery bypass grafting: Are traditional risk scores accurate? PLoS ONE 16(8): e0255662. https://doi.org/10.1371/journal.pone.0255662
