# Supplementary material for: Mortality risk prediction in high-risk patients undergoing coronary artery bypass grafting: Are traditional risk scores accurate?
Source: PLoS One. 2021 Aug 3;16(8):e0255662. doi: 10.1371/journal.pone.0255662 (PMC8330943; doi:10.1371/journal.pone.0255662)
Supplement: S1 Table — (DOCX) [file pone.0255662.s001.docx]

**S1 Table.** Characteristics of the final sample of patients from Brazil and China submitted to isolated CABG with ESII between 5-10%

|  | Brazil (n = 222) | | China (n = 26) | | Total (n = 248) | | | p |
| --- | --- | --- | --- | --- | --- | --- | --- | --- |
|  | **n** | **%** | **n** | **%** | **n** | **%** | **95% CI** |  |
| Age (years) | 69,96 ± 9,0 | | 66,42 ± 12,56 | | 69,9 ± 9,45 | | 68.55–70.90 | 0.305 |
| Body mass index | 26,38 ± 4,7 | | 23,8 ± 1,9 | | 26,11 ± 4,55 | | 25.50–26.60 | 0.001 |
| SPAP (mmHg) | 27,6 ± 13,7 | | 24,2 ± 8,7 | | 27,3 ± 13,3 | | 25.70–29.00 | 0.243 |
| LVEF (%) | 45,1 ± 14,9 | | 53,4 ± 11,8 | | 46,0 ± 14,8 | | 43.90–47.60 | 0.002 |
| Serum glucose (mg/dL) | 154,4 ± 80,9 | | 137,0 ± 56,3 | | 152 ± 78 | | 140.7–161.16 | 0.345 |
| Creatinine (mg/dL) | 1,55 ± 1,15 | | 1,3 ± 0,38 | | 1,52 ± 1,1 | | 1.37–1.64 | 0.467 |
| EuroSCORE II | 6,66 ± 1,3 | | 6,49 ± 1,24 | | 6,6 ± 1,3 | | 6.47–6.80 | 0.619 |
| Gender (male) | 107 | 48.2 | 12 | 46.2 | 119 | 47.98 | 0.42–0.54 | 0.844 |
| Arterial hypertension | 194 | 87.4 | 18 | 69.2 | 212 | 85.48 | 0.80–0.90 | 0.013 |
| Peripheral artery disease | 47 | 21.2 | 2 | 7.7 | 49 | 19.76 | 0.15–0.25 | 0.102 |
| Dialysis | 4 | 1.8 | 0 | 0.0 | 4 | 1.61 | 0.004–0.40 | 0.49 |
| Cerebrovascular disease | 66 | 29.7 | 7 | 26.9 | 73 | 29.44 | 0.24–0.36 | 0.776 |
| IABP before surgery | 43 | 19.4 | 7 | 26.9 | 50 | 20.16 | 0.15–0.26 | 0.364 |
| Pulmonary hypertension | 138 | 62.2 | 6 | 23.1 | 144 | 58.06 | 0.51–0.64 | 0.000 |
| Diabetes mellitus | 132 | 59.5 | 11 | 42.3 | 143 | 57.66 | 0.51–0.63 | 0.094 |
| Insulin-dependent | 72 | 32.4 | 8 | 30.8 | 80 | 32.26 | 0.26–0.38 | 0.864 |
| COPD | 58 | 26.1 | 1 | 3.8 | 59 | 23.79 | 0.19–0.29 | 0.012 |
| Atrial fibrillation |  |  |  |  |  |  |  |  |
| Persistent/paroxysmal | 16 | 7.2 | 0 | 0.0 | 16 | 6.45 | 0.05–0.13 | 0.261 |
| Continued/permanent | 5 | 2.3 | 0 | 0.0 | 5 | 2.02 |  |  |
| Previous cardiac intervention |  |  |  |  |  |  |  |  |
| Previous PCI | 43 | 19.4 | 0 | 0.0 | 43 | 17.34 | 0.13–0.23 | 0.014 |
| Previous CABG | 7 | 3.2 | 2 | 7.7 | 9 | 3.63 | 0.02–0.07 | 0.242 |
| Previous valve procedure | 4 | 1.8 | 0 | 0.0 | 4 | 1.61 | 0.004–0.40 | 0.490 |
| Previous MI | 171 | 77.0 | 13 | 50.0 | 184 | 74.19 | 0.69–0.79 | 0.003 |
| Coronary disease | 222 | 100.0 | 21 | 80.8 | 245 | 98.79 | 0.95–0.99 | 0.000 |
| Three-vessel coronary artery disease | 186 | 83.8 | 15 | 57.7 | 201 | 81.05 | 0.76–0.86 | 0.001 |
| Left main disease > 50% | 74 | 33.3 | 1 | 3.8 | 75 | 30.24 | 0.25–0.36 | 0.002 |
| NYHA I | 39 | 17.6 | 1 | 3.8 | 40 | 16.13 | - | - |
| NYHA II | 37 | 16.7 | 1 | 3.8 | 38 | 15.32 | - | - |
| NYHA III | 62 | 27.9 | 13 | 50.0 | 75 | 30.24 | - | - |
| NYHA IV | 52 | 23.4 | 11 | 42.3 | 63 | 25.40 | 0.49–0.62 | - |
| CCS 4 | 99 | 44.6 | 24 | 92.3 | 123 | 49.60 | 0.43–0.56 | 0.000 |
| Moderate heart valve disease | 72 | 32.4 | 18 | 69.2 | 90 | 36.29 | 0.30–0.42 | 0.000 |
| Urgency/emergency status | 186 | 83.8 | 26 | 100.0 | 212 | 85.48 | 0.80–0.89 | 0.026 |
| Death | 30 | 13.5 | 3 | 11.5 | 33 | 13.31 | 0.09–0.18 | 0.779 |

CABG: coronary artery bypass grafting; CCS: Canadian Cardiovascular Society; COPD: chronic obstructive pulmonary disease; IABP: intra-aortic balloon pump; LVEF: left ventricle ejection fraction; MI: myocardial infarction; NYHA: New York Heart Association; PCI: percutaneous coronary intervention; SPAP: systolic pulmonary artery pressure.
